# Influence of dynamic stretching on ankle joint stiffness, vertical stiffness and running economy during treadmill running

**DOI:** 10.3389/fphys.2022.948442

**Published:** 2022-10-06

**Authors:** George M. Pamboris, Marika Noorkoiv, Vasilios Baltzopoulos, Douglas W. Powell, Tom Howes, Amir A. Mohagheghi

**Affiliations:** ^1^ Department of Health Sciences, School of Sciences, European University Cyprus, Nicosia, Cyprus; ^2^ Division of Sport, Health, and Exercise Sciences, Brunel University London, Uxbridge, United Kingdom; ^3^ Research Institute for Sport and Exercise Sciences (RISES), Liverpool John Moores University, Liverpool, United Kingdom; ^4^ School of Health Studies, University of Memphis, Memphis, TN, United States

**Keywords:** running economy, joint stiffness, vertical stiffness, kinetics, biomechanics, dynamic stretching

## Abstract

The purpose of the present study was to investigate whether and how dynamic stretching of the plantarflexors may influence running economy. A crossover design with a minimum of 48 h between experimental (dynamic stretching) and control conditions was used. Twelve recreational runners performed a step-wise incremental protocol to the limit of tolerance on a motorised instrumented treadmill. The initial speed was 2.3 m/s, followed by increments of 0.2 m/s every 3 min. Dynamic joint stiffness, vertical stiffness and running kinematics during the initial stage of the protocol were calculated. Running economy was evaluated using online gas-analysis. For each participant, the minimum number of stages completed before peak O_2_ uptake (V̇O_2peak_) common to the two testing conditions was used to calculate the gradient of a linear regression line between V̇O_2_ (*y*-axis) and speed (*x*-axis). The number of stages, which ranged between 4 and 8, was used to construct individual subject regression equations. Non-clinical forms of magnitude-based decision method were used to assess outcomes. The dynamic stretching protocol resulted in a *possible* decrease in dynamic ankle joint stiffness (−10.7%; 90% confidence limits ±16.1%), a *possible* decrease in vertical stiffness (−2.3%, ±4.3%), a *possibly beneficial* effect on running economy (−4.0%, ±8.3%), and *very likely* decrease in gastrocnemius medialis muscle activation (−27.1%, ±39.2%). The results indicate that dynamic stretching improves running economy, *possibly via* decreases in dynamic joint and vertical stiffness and muscle activation. Together, these results imply that dynamic stretching should be recommended as part of the warm-up for running training in recreational athletes examined in this study.

## Introduction

Pre-warm-up activities are widely used in sports events to prepare the body for optimum performance (Bishop, 2003). Stretching is usually included as part of the warm-up, but the effect of different types of stretching on aspects of performance and the mechanisms through which they induce their proposed effects are less clear. For example, comparing different stretching techniques, Behm et al. (2015) reported mean performance impairments in strength tasks of 3.7% and 4.4% immediately after static and proprioceptive neuromuscular facilitation (PNF) stretching, respectively, but an increase in performance of 1.3% after dynamic stretching. Dynamic stretching is one of the most common pre-warm-up activities used by distance runners because it is likely to induce beneficial cardiovascular changes contributing to running performance (Bishop, 2003). More recent research has also focused on biomechanical and other physiological mechanisms that may be affected by dynamic stretching and influence running performance.

It has been suggested that biomechanical and physiological properties of the muscle-tendon unit (MTU) and/or its individual components, such as muscle or tendon stiffness, and overall joint mechanics can be affected by dynamic stretching. This, in turn, may alter physiological variables such as oxygen uptake, V̇O_2_ kinetic responses, lactate threshold, (Moore, 2016), hence affecting running economy and endurance performance (Arampatzis et al., 2006; Kubo et al., 2010a; Kubo et al., 2010b; Hunter et al., 2011; Fletcher et al., 2013b; Kubo et al., 2015a; Kubo et al., 2015b). For information on the different types of stiffness, the reader can refer to Butler et al. (2003) and Brughelli and Cronin (2008).

To support this view, some studies (Arampatzis et al., 2006; Albracht and Arampatzis, 2013) reported that more economical runners (i.e., those with lower V̇O_2_ for a given running speed) display a greater plantarflexor muscle strength and triceps surae tendon stiffness due to reduced energy requirements of the muscles. Kyröläinen and Komi (1994) found that stiffer muscles surrounding the ankle and knee joints caused force potentiation when transitioning from the braking to the propulsion phase of running, thereby improving running economy. On the contrary, it may be argued that a more compliant tendon will store more elastic energy, which can be released during the positive work phase of the lower limb musculature involved in locomotion (Cavagna et al., 1988), while a stiffer tendon and aponeurosis would increase the metabolic energy cost during the propulsion phase of running. Indeed, a number of studies reported that lower leg stiffness was related to superior running performance as assessed by personal best time and running economy (Kubo et al., 2010a; Kubo et al., 2010b; Kubo et al., 2015b). In general, these proposed mechanisms for the effect of stretching on running economy are based around the stretching effect on the bouncing of body centre of mass (COM) during running, which has been characterised by the vertical stiffness of a spring-mass system, reflecting the stiffness of the entire lower limb (McMahon and Cheng, 1990). This suggests that training methods may determine vertical stiffness, potentially modifying the role of the elastic and contractile components of the MTUs during running.

There are other mechanisms *via* which dynamic stretching can affect running economy. In addition to increasing flexibility (range of motion), dynamic stretching decreases passive joint stiffness (Herda et al., 2013) and/or increases motor unit activation (Cramer et al., 2004; Torres et al., 2008; Hough et al., 2009; Fletcher, 2013). Increased flexibility and motor unit activation can alter the SSC performance, which in turn may affect running economy. For a task involving SSC, an optimal tendon stiffness exists for power output and efficiency (Alexander and Bennet-Clark, 1977; Butler et al., 2003). Subsequently, stretching to increase flexibility may result in less than optimal joint mechanical properties during performance whereby muscle energy requirement is minimised (Fletcher et al., 2013a). Another possible mechanism for lower flexibility contributing to higher running economy is the association of running economy with shorter muscle fascicles and shorter muscles that use less energy when the velocity of shortening is not important (Fletcher and MacIntosh, 2017). Therefore, there is a delicate balance between the flexibility of the joints, MTU stiffness and muscle activation after dynamic stretching and physiological responses of the performer as determined by the running economy.

Only a few studies have examined the effects of dynamic stretching on running economy. Hayes and Walker (2007) compared the acute effects of controlled-velocity dynamic stretching on running economy at 75% V̇O_2_max in well-trained distance runners. The authors found that slow velocity dynamic stretching did not acutely change running economy. Zourdos et al. (2012) showed that dynamic stretching of the lower extremities was related to an increase in the energy (caloric) expenditure during treadmill running for 30 min at 65% V̇O_2_max. Yamaguchi et al. (2015) investigated the effects of dynamic stretching at a velocity equivalent to 90% V̇O_2_max. Dynamic stretching increased the time to exhaustion but did not affect running economy; however, the time to exhaustion and running distance were prolonged in the dynamic stretching group compared to those in the non-stretching control. Yamaguchi et al. (2015) argued that the differences between these studies’ findings were mainly due to differences in the protocols and exercise intensities during performance assessment. However, the underlying mechanisms for improved performance are not known, and it is unclear whether a change in MTU stiffness and/or improvement in neuromuscular activation was altered by dynamic stretching and caused the above effects on running economy. It has been suggested that a dynamic warm-up may increase flexibility due to an increase in muscle compliance, whereas static stretching increases flexibility due to an increase in MTU compliance, causing an increase in the slack of both muscle and tendon creep, which in turn impairs subsequent performance (Carter and Greenwood, 2015).

While it is proposed that dynamic stretching decreases passive MTU stiffness, its influence on joint and overall vertical stiffness and associated kinematic and kinetic variables during running, as possible mechanisms through which running economy can be affected, is not known. The purpose of this study was to examine the acute effect of dynamic stretching on running economy and determine the underlying mechanisms through which running economy can be affected.

## Materials and methods

### Participants

Twelve healthy male recreational runners (mean ± standard deviation; age: 27.3 ± 4.4 years; height: 1.80 ± 0.05 m; body mass: 70.0 ± 0.1 kg) with no history of musculoskeletal injury during the previous 6 months volunteered for the study. All participants were familiar with running on a treadmill. Each participant was tested at a similar time (14:00) of the day (±1 h) and was asked to maintain a consistent lifestyle (similar diet and no unaccustomed exercise) between visits. Participants were asked to eat or drink 2 h before the experiments. They were instructed to report to the laboratory well hydrated, rested, and completed no strenuous exercise within the previous 48 h before the testing sessions. They were prohibited from drinking alcohol or caffeine within the last 24 h and 6 h, respectively. The study was approved by the Brunel University London Research Ethics Committee (application code: RE26–14).

### Testing overview

The participants visited the laboratory on two occasions, separated by ≥ 48 h but completed within the period of 2 weeks to avoid any potential carryover effects (Guglielmo et al., 2009). At the first visit, the participants undertook either dynamic stretching (experimental condition) or no stretching (control) in a randomised, counterbalanced order. Each participant wore the same shoes during both conditions.

### Anthropometry

Prior to each trial, body mass was measured to the nearest 0.1 kg using an analogue balance scale (Seca Vogel & Halke GmbH & Co., Hamburg, Germany), and height was recorded to the nearest 1 cm using a stadiometer (Marsden Leicester Height Measure, Marsden Weighing Group, Rotherham, United Kingdom).

### Interventions

**FIGURE 1:**
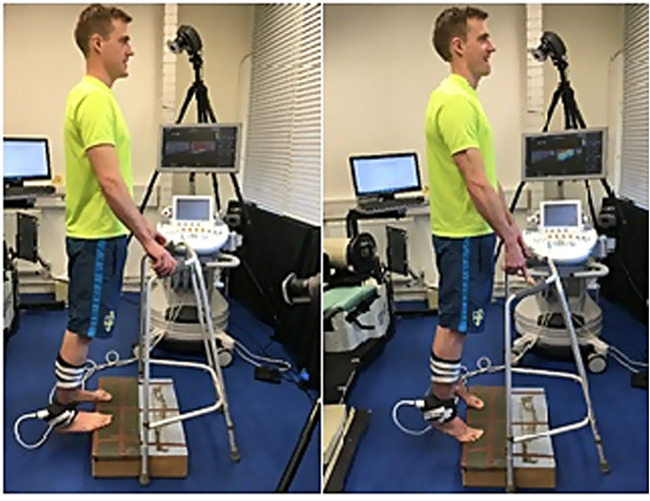
Start and finish position **(A)**. Position at full stretch **(B)**.

For the dynamic stretching protocol each participant stood on a step that was approximately 20 cm in height, and started on the balls of both feet with the heels raised and then lowered in a controlled manner (Figure 1). The stretching exercise was performed on the edge of the step, and participants were instructed to move into full plantarflexion and dorsiflexion during the protocol. The stretching exercise was performed at 100 beats/min (MetroTimer 3.3.2, ONYX three Apps, Sofia, Bulgaria), and participants completed three sets of 20 repetitions with 5 s rest between sets. Ankle plantarflexors (Gastrocnemius medialis (GM) and soleus (SOL)) were the target muscles for the stretching protocol since during both active plantarflexion (concentric contraction of GM and SOL) and dorsiflexion (eccentric contraction of the GM and SOL), contraction of the “agonist” muscle group ankle plantarflexors was ensured. The control condition involved participants sitting quietly for 50 s, which was equivalent to the duration of the dynamic stretching protocol.

### Kinematic and kinetic analyses

**FIGURE 2:**
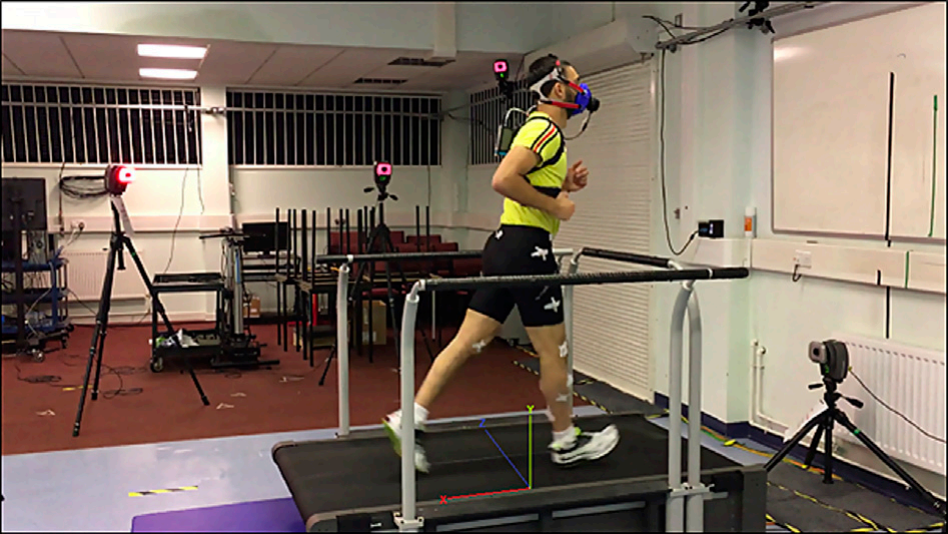
Laboratory set-up with the lab-coordinate system shown on the treadmill.

Joint kinematics during running were monitored using a 10-camera motion-capture system (Motion Analysis Corporation Inc. Santa Rosa, CA, United States) synchronised with a treadmill with dual integrated force plates capable of capturing three-dimensional force components (Bertec Corporation, Columbus, OH, United States) (Figure 2).

Spherical retro-reflective markers were placed on the surface of the body over the following anatomical landmarks: sacrum (mid-posterior superior iliac spine (PSIS), and bilaterally on the anterior superior iliac spine (ASIS)), greater trochanter, lateral femoral epicondyle, medial femoral epicondyle, lateral malleolus, medial malleolus, shoe at the location of the heel (calcaneus), and first and fifth metatarsal heads. A calibration trial was conducted prior to testing where each participant was asked to adopt the upright standing position on the treadmill and a neutral position (baseline) was recorded while the participant stood upright. Tracking markers were placed on both thighs and shanks. Motion data were collected at 150 Hz, and ground reaction forces were obtained at 2,100 Hz. Participants ran at set speeds according to the maximum incremental exercise test (see below). Five running trials were collected, and the mean of these cycles was used in the analysis. Joint angles were calculated using a right-hand rule with Cardan rotational X-Y-Z sequence to describe the motions of the distal segment relative to the proximal segment. Rotations about the *Z*-axis corresponded to flexion/extension, about the *X*-axis to abduction/adduction, and about the *Y*-axis to internal/external rotation.

Joint angles were computed as the angles between the proximal and distal segment of the relevant joint. The line connecting the segments was calculated by finding the midpoint between medial and lateral markers. Specifically, ankle angle was measured as the angle between the line connecting the malleoli and the metatarsal heads (i.e., foot segment), and the line connecting the malleoli and the epicondyles (i.e., lower leg segment). Ankle sagittal joint angle was calculated as the deviation from the upright standing posture (neutralposition) and used to define plantarflexion/dorsiflexion by subtracting the ankle angle value at the standing position from the raw kinematic data. Joint kinematics were calculated using inverse kinematics, and net joint moments were calculated using a standard inverse dynamics approach and normalised to body mass using dedicated software (Visual 3D, C-motion, Germantown, MD, United States). The external moments acting on the ankle joint were expressed in the ankle joint coordinate system of the anatomical model as this has been suggested to be the best option for a standardised system as it represents what a joint moment actually is (Schache and Baker, 2007). Gait events were determined using force platform data from heel strike (initial contact) to toe-off to enable calculation of kinematic, ankle moment and EMG data during the stance phase of gait. The force platform system was factory-calibrated, with manual zeroing performed when no load was acting on the force plates.

Marker trajectories were low-pass filtered at 6 Hz, and ground reaction force data were filtered at 30 Hz using a fourth order zero-lag Butterworth low-pass filter. Each stance phase of the gait cycle (time points between heel strike to toe-off) was time normalised to 101 points (Zeni et al., 2008). Heel strike events were determined when the vertical ground reaction force crossed a threshold of 20 N for a period of at least 0.05 s, whereas toe-off was defined as the point after initial contact at which the vertical ground reaction force fell below 20 N for a period of at least 0.05 s. All kinematic and kinetic variables of interest for the ankle, including peak angles, maximum range of motion (ROM) and peak joint moments, were calculated for the right leg for all participants.

Dynamic joint stiffness was defined as the change in joint moment normalised to body mass divided by the change in joint angle and was calculated as the slope of the ankle moment-angle plot during the propulsion phase of stance (loading response to toe-off). Vertical stiffness was calculated as the peak vertical ground reaction force divided by the vertical displacement of the pelvis between initial contact and peak vertical ground reaction force. Vertical stiffness was corrected by multiplying the calculated values by a correction factor 1.0496 (Coleman et al., 2012) to reduce systematic error. For all biomechanical variables, the mean of five running cycles at the initial running speed collected after 30 s into the first running stage was used in the statistical analysis.

Custom-made software (Matlab 2013a; MathWorks, Natick, MA, United States) was used to calculate dynamic joint stiffness and vertical stiffness using the original dataset (i.e., not time normalised) (Farley and Morgenroth, 1999; Powell et al., 2014).

### Exercise protocol

Participants started running on the treadmill after 2 min of dynamic stretching or control, which represents the minimum period between warm-up and start of a game/training session, used by previous authors (Fletcher and Jones, 2004). The participants performed a step-wise incremental protocol to the limit of tolerance. The initial speed was 2.3 m/s, followed by increments of 0.2 m/s every 3 min.

### Online gas-analysis

Pulmonary gas-exchange was measured for 6 min at rest (baseline) and continuously during exercise using a portable metabolic system (Cosmed K5, Rome, Italy) that incorporated gas analysers (O_2_ and CO_2_) and a bidirectional flow turbine. The gas analysers were calibrated before each test using ambient air and a gas mixture of known concentration (5% CO_2_, 16% O_2_, balance N_2_) according to the manufacturer’s recommendations. The turbine was calibrated with a 3 L syringe (Cosmed Srl, Rome, Italy). The gas-concentration and volume signals were time-aligned, thereby accounting for the transit delay in capillary gas and analyser rise-time relative to the volume signal. The equipment was positioned on the participant, and the bidirectional turbine was attached to a facemask (Hans Rudolph Inc. Shawnee, KS, United States) covering both the mouth and the nose. Participants breathed through the low-dead-space mask, with air sampled at 200 ml min^−1^. Breath-by-breath V̇O_2_ data were initially examined to exclude errant breaths caused by coughing, swallowing, *etc.*, and breath-by-breath data were averaged over 5 breaths. Any values lying more than three SD from the local mean were removed. V̇O_2,_ carbon dioxide output (V̇CO_2_) and respiratory exchange ratio (RER) were quantified over the final 30 s of each stage of the step-wise incremental protocol.

### Calculation of running economy

Participants remained seated and still in order to obtain metabolic cost measures for rest. While running, the 30 s average V̇O_2_ was determined during the final minute of each stage. Running economy was established by plotting V̇O_2_ (*y*-axis) vs. speed, with the method of least squares used to establish the extent to which the data conformed to the expected linear relationship and the production of a linear regression equation (Di Prampero et al., 2009). For each participant, the minimum number of whole stages completed before V̇O_2peak_ was achieved in both conditions was used. The number of stages used to construct individual subject regression equations ranged from 4 to 8. This was found to be accurate (Control: *R* ^2^, 0.96; SEE, 0.088 ml kg^−1^ min^−1^; Dynamic stretching: *R* ^2^, 0.94; SEE, 0.097 ml kg^−1^ min^−1^). To investigate the time course of change in V̇O_2_ over the range of speeds/numbers of stages completed by participants, we included the stages completed by all participants.

### Electromyography

Surface electromyography (EMG) was performed using three Trigno Wireless electrode sensors (Delsys Inc. Ltd. Boston, United States), which had a predetermined bandwidth of 20–450 Hz, a gain of 1,000, a common-mode rejection ratio of >80 dB, and a sampling rate of 2,000 Hz. After standard skin preparation, the electrodes were placed over the MG, SOL and Tibialis anterior (TA) according to SENIAM guidelines (Hermens et al., 1999). The EMG signals were collected at a sampling rate of 2,100 Hz and stored for offline analysis. All EMG data were visually inspected prior to analysis. EMG data for each gait cycle were exported from Visual 3D and imported into Spike2 software (Cambridge Electronic Design, Cambridge, United Kingdom) for further analysis. EMG raw signals were notch filtered at 50 Hz (to remove ambient noise from power supply), rectified and smoothed using a 5-point moving average (Spike2, Cambridge Electronic Design, Cambridge, United Kingdom). The EMG amplitude for each muscle was calculated as root mean square (RMS) over five stance cycles (initial contact to toe-off).

### Statistical analysis

Sample size was calculated using the method of Hopkins (Hopkins, 2006a). Assuming a between-subject coefficient of variation in V̇O_2_ of 13.8%, a within-subject standard deviation (typical error) of 0.45% (Shaw et al., 2013), and chances of type I and type II errors of 0.5% and 25%, respectively. For these numbers, the lowest sample size (50% intervention group/50% control group) was 10/10, which was surpassed in the present study (12/12). Descriptive statistics were reported as means and SDs. Data analysis was undertaken using a post-only crossover trial with adjustment for a predictor spreadsheet (Hopkins, 2006b). The effect size, which represents the differences between conditions, was calculated from log-transformed and subsequently back-transformed data, with 90% CI reported as estimates of uncertainty to quantify the magnitude of the difference between pre-intervention and post-intervention outcome performance measures (Hopkins et al., 2009). This is suggested to be the appropriate method for quantifying changes in athletic performance (Hopkins et al., 2009). Dependent variables were analysed either as log-transformed data (all physiological measures, ankle joint stiffness, vertical stiffness, moments and EMG amplitudes) or raw data (ROM, angles) (Hopkins, 2015). The threshold value for the smallest worthwhile change (SWC) was set at 0.2 of the between-subject deviation for all measures with the rationale being that the current exploration of these effects is novel so we have no *a priori* information on physiological effect sizes and the probability that the true value of the effect was greater than the SWC was calculated and interpreted qualitatively. Qualitative magnitudes of observed effects were assessed *via* standardization with the following scale for Cohen’s d (fractions and multiples of the baseline standard deviation) and wereclassified as trivial (<0.2), small (0.2–0.6), moderate (0.6–1.2), large (1.2–2.0), very large (2.0–4.0) or extremely large (>4.0) (Hopkins et al., 2009; Hopkins, 2019b). Uncertainty was presented as 90% compatibility interval (CI) (Gelman and Greenland, 2019). In keeping with recent calls to advance statistical analysis and reporting (Amrhein et al., 2019), inference was based on probabilistic decisions about true (large sample) magnitudes based on two one-sided hypothesis tests of substantial (at least small) effects followed by Bayesian inference (Hopkins et al., 2009). The approach sits within the inferential family of equivalence, non-inferiority and minimal effects or superiority testing (Piaggio et al., 2012). The *p* value for rejecting a hypothesis of a given substantial magnitude was the area of the sampling t distribution of the effect statistic of that magnitude (Lakens et al., 2018). Hypotheses of inferiority (substantial negative) and superiority (substantial positive) were rejected if their respective *p* values were <0.05; rejection of both hypotheses represents a decisively trivial effect in equivalence testing. If neither hypothesis was rejected, the magnitude of the observed effect was considered to be unclear. When only one hypothesis was rejected, the *p* value for the other hypothesis, when >0.25, was interpreted as the posterior probability of a substantial true magnitude of the effect in a reference-Bayesian analysis with a minimally informative prior (Hopkins and Batterham, 2018; Hopkins, 2019a) using the following scale: <0.5%, most unlikely or almost certainly not; 0.5–5%, very unlikely; 5%–25%, unlikely or probably not; 25%–75%, possibly; 75%–95%, likely or probably; 95%–99.5%, very likely; and >99.5%, most likely or almost certainly (Hopkins et al., 2009).

## Results

The before and after stretching values (mean ± standard deviation) with mean differences, effect size and qualitative non-clinical inferences based on post-only crossover trial analysis are shown in Table 1.

**TABLE 1:** Descriptive statistics (mean ± SD) and mean differences in the control and dynamic stretching (DS) outcome measures along with effect sizes and qualitative inferences.

	Control	DS	Mean change; 90% CL	Effect size	Likelihood (%) of DS being beneficial/trivial/detrimental	Qualitative inference
Physiological Measures						
V̇O_2_– *y*-intercept (m/kg/min)	35.24 ± 5.52	35.30 ± 3.99	+0.8 ± 3.3	+0.04 ± 0.18	7/91/2	Likely trivial
V̇O_2_ (1st stage) (mL/kg^−^/min^/^)	39.53 ± 4.29	38.59 ± 4.23	-2.0 ± 4.3	-0.16 ± 0.36	5/52/43	Possibly beneficial
V̇O_2_ (2nd stage) (ml/kg/min)	42.95 ± 5.41	42.85 ± 5.36	0.0 ± 2.6	0.0 ± 0.19	4/91/4	Likely trivial
V̇O_2_ (3rd stage) (ml/kg/min)	46.65 ± 5.32	46.52 ± 5.62	4.0 ± 26.2	0.31 ± 2.00	54/14/32	Unclear
V̇O_2_ (4th stage) (ml/kg/min)	49.11 ± 5.50	48.57 ± 5.81	4.7 ± 31.6	0.38 ± 2.45	55/11/34	Unclear
Biomechanical Measures						
Joint Stiffness (Nm·kg^−1^·deg.^−1^)	0.067 ± 0.030	0.060 ± 0.033	-10.7 ± 16.1	-0.20 ± 0.31	2/49/49	Possibly decrease
Vertical Stiffness (N·m^−1^)	23,195 ± 2,483	22,708 ± 2,990	-2.3 ± 4.3	-0.20 ± 0.37	4/46/50	Possibly decrease
Peak Plantarflexion Moment (Nm·kg^−1^)	1.62 ± 0.14	1.68 ± 0.18	+2.1 ± 5.7	+0.22 ± 0.60	53/36/12	Unclear
Dynamic ROM (deg.)	32.18 ± 3.65	31.88 ± 3.82	-0.3 ± 1.0	-0.08 ± 0.27	5/54/21	Unlikely decrease
Peak Plantarflexion Angle (deg.)	-14.00 ± 4.16	-15.36 ± 5.27	-1.4 ± 2.2	-0.30 ± 0.49	5/31/64	Possibly increase
Peak Dorsiflexion Angle (deg.)	18.16 ± 2.84	16.81 ± 4.83	-1.4 ± 2.4	-0.44 ± 0.78	8/21/71	Unclear
Ankle Angle at Initial Contact (deg.)	5.37 ± 3.59	3.24 ± 5.77	-2.1 ± 2.5	-0.55 ± 0.66	3/15/82	Likely decrease
Ankle Angle at Toe-Off (deg.)	-13.93 ± 4.27	-14.79 ± 5.55	-0.9 ± 2.2	-0.19 ± 0.48	9/43/48	Unclear
EMG RMS (GM) (V)	0.18 ± 0.25	0.10 ± 0.04	-27.1 ± 39.2	-0.38 ± 0.15	0/3/97	Very likely decrease
EMG RMS (SOL) (V)	0.12 ± 0.06	0.11 ± 0.04	-16.3 ± 20.9	-0.23 ± 0.49	7/39/54	Unclear
EMG RMS (TA) (V)	0.08 ± 0.03	0.07 ± 0.04	-8.6 ± 19.6	-0.19 ± 0.40	6/49/44	Unclear

*Note.* Physiological and biomechanical measures are reported as log-transformed data, except for peak plantarflexion moment dynamic ROM, peak ankle and dorsiflexion angles, angles at initial contact and toe-off, which are reported as raw data.

### Physiological measures

**FIGURE 3:**
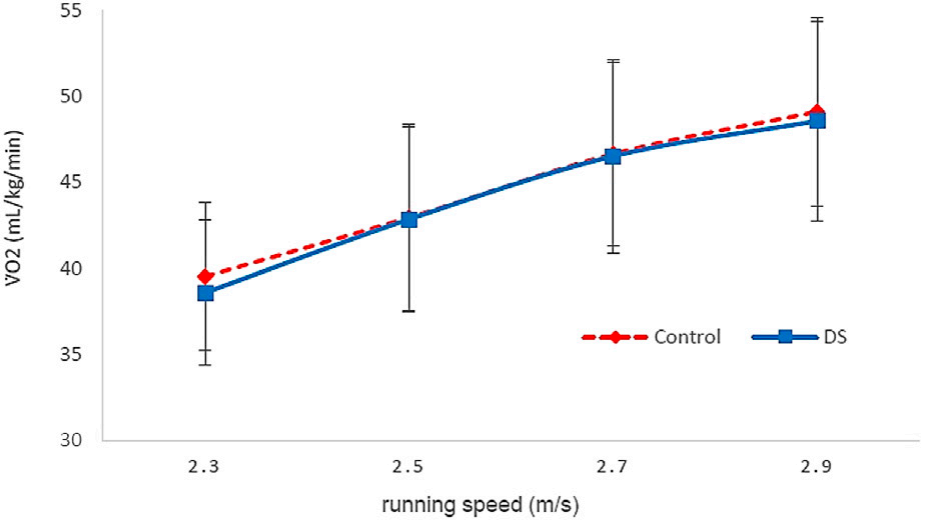
V̇O_2_ vs. running speed for Dynamic stretching (DS) and Control (C) over the first four stages. Error bars are standard deviations.

Dynamic stretching resulted in a likely trivial change in the *y*-intercept (0.04% ± 0.18, trivial effect). In response to dynamic stretching, there was a possible beneficial change in V̇O_2_ during the first stage of the protocol (−2.0% ± 4.3; trivial effect), indicating increased running economy, a likely trivial effect (0.0% ± 2.6, trivial effect) indicating no change on running economy at the second stage and unclear effects at the third and fourth stage (Figure 3).

### Biomechanical measures

Dynamic stretching resulted in a possible decrease in joint stiffness (−10.7% ± 16.1; small effect) and a possible decrease in vertical stiffness (−2.3% ± 4.3; small effect). The effects of dynamic stretching on plantarflexion moment (normalised to body mass) were unclear. Dynamic stretching intervention resulted in an unlikely decrease in dynamic ROM (-0.3° ± 1.0; trivial effect).

There was a very likely decrease in ankle angle at initial contact (−2.1° ± 2.5; moderate effect), whilst the effect on ankle angle at toe-off was unclear (Table 1). Dynamic stretching resulted in a possible increase in peak ankle plantarflexion angle (−1.4° ± 2.2; small effect) and an unclear effect on peak ankle dorsiflexion angle.

There was a very likely decrease in GM RMS (-27.1% ± 9.2; small effect), whilst there was a decrease in both SOL and TA RMS, although this effect was unclear.

## Discussion

This study aimed to investigate the potential effects and underlying mechanisms of an acute bout of dynamic stretching on running economy during a maximal incremental exercise test. Dynamic stretching elicited a *possibly* better overall running economy and reduction of V̇O_2_ during the first stage of running, and a likely trivial effect during the second stage, but the effect during the third and fourth stages was unclear. This suggests that the time course of the effect of dynamic stretching was short but enough to possibly decrease the gradient of the V̇O_2_ -speed regression line. The data also revealed that dynamic stretching possibly decreased ankle joint and vertical stiffness during running at 2.3 m/s (first stage). Ankle plantarflexion ROM during running was larger after dynamic stretching, thereby providing a mechanistic explanation for the observed lower dynamic ankle joint stiffness. The results are in agreement with those of Kubo et al. (2010b), who found that those participants with decreased tendon stiffness had better running economy. Furthermore, the results suggest that an acute dynamic stretching protocol could alter lower extremity joint kinematics and kinetics and physiology of neuromuscular functions during running. To our knowledge, this is the first study to demonstrate an improvement on running economy following a dynamic stretching protocol and investigate the possible mechanisms behind it. However, this study did not establish a relationship between dynamic joint stiffness, vertical stiffness and running economy since physiological and biomechanical data were not collected synchronously (measurements were collected in two different moments in time).

The physiological findings were in contrast with other studies (Hayes and Walker, 2007; Zourdos et al., 2012; Yamaguchi et al., 2015). Hayes and Walker (2007) compared the effects of five exercises of dynamic and static stretching on running economy during 10 min of constant-speed treadmill running at 75% V̇O_2_max, and found no change on running economy. They attributed their results to the inclusion of a 10-min submaximal warm-up run prior to the testing, which could have reversed the reductions in neuromuscular performance and nullified the stretching effect. Moreover, Yamaguchi et al. (2015) examined the effect of dynamic stretching on five lower limb muscles of one set of 10 repetitions performed as quickly as possible and found no effect on oxygen uptake at 90% V̇O_2_max. In contrast, Zourdos et al. (2012) found an increase in energy expenditure by 4.4% during treadmill running after the dynamic stretching intervention (10 exercises, 2 sets × 4 repetitions) compared to the non-stretching controls at an intensity of 65% V̇O2max for 30 min. The dynamic stretching raised the participants’ resting heart rate, V̇O_2_, and metabolism before the run. However, this temperature and metabolic demand increase may not be beneficial to endurance performance (Bishop, 2003). Performance may suffer if the warm-intensity up is too high or lasts for too long (>10 min) (Bishop, 2003). It is possible that the longer dynamic stretching protocol duration (900 s) (Zourdos et al., 2017) was excessive and negatively affected the results (Konrad et al., 2021). The warm-up should increase V̇O_2_ while avoiding premature fatigue and increasing body temperature (Zourdos et al., 2017). Possible reasons for the differences between the outcomes of the above studies and the present study are discussed below.

Firstly, the dynamic stretching protocols used in the previous studies (Hayes and Walker, 2007; Zourdos et al., 2012; Yamaguchi et al., 2015) differed from the current study in terms of repetitions, sets and the velocity of dynamic stretching. One study (Hayes and Walker, 2007) used slow-velocity dynamic stretching, and the other study (Zourdos et al., 2012) used a small volume of dynamic stretching. Another previous study (Yamaguchi et al., 2015) utilised dynamic stretching for 10 repetitions as quickly as possible as part of the protocol. Konrad et al. (2021) recommend that an optimal dynamic stretching for a short duration of up to 220 s in total is performed. Secondly, there were differences in the exercise intensities when assessing running economy. In the current study, for each participant, all stages of the run were completed before V̇O_2_ peak was chosen for the analysis. Comparing baseline V̇O_2_ (*y*-intercept) at the start of the incremental treadmill exercise test between stretching and control conditions, we found that dynamic stretching only caused a likely trivial change in the baseline oxygen consumption. Therefore, we can reject that elevated oxygen consumption before the running task was a contributing factor to the improved performance as suggested by Bishop (2003).

The other mechanism(s) causing the change on running economy is thought to be a change in the properties of the MTU and/or a change in motor unit activation as a consequence of the stretching procedure (Fletcher and Anness, 2007). This is supported by the current results, which suggest a small decrease in the joint and vertical stiffness as a contributor to the possibly improved running economy. Recently, Pappas et al. (2017) investigated the effects of dynamic stretching on vertical stiffness during running and showed that vertical stiffness was not altered, which contradicts the current findings. Herda et al. (2013) reported a decrease in MTU stiffness following four 30 s sets of dynamic stretching, which agrees with the current findings. One possible reason for the discrepancy in the stiffness effect between the present study and Pappas et al. (2017) may be the inclusion of a different dynamic stretching protocol in their study (5-min warm-up at 2.22 m/s, number of lower leg muscles stretched, duration of stretching on each muscle-two 20 s bouts, and the higher running speed 4.44 m/s) in contrast with the protocol of the current study.

There are other physiological factors associated with a more active warm-up (Fletcher, 2010) that can benefit performance. Another possible reason for the enhanced performance following dynamic stretching may be the rehearsal of more task-specific movement patterns of our protocol, such as dorsiflexion-plantarflexion, which match the movement patterns and velocity of the running task (Fletcher and Jones, 2004). Results from the EMG analysis indicated that dynamic stretching caused a small decrease in GM RMS during the gait cycle, and although the effects on Sol and TA RMS values were unclear, there was a tendency for a decrease. The less muscle activation for running at a given speed would reduce energy expenditure improving running economy (Tartaruga et al., 2012). Although not controlled in this study, rehearsal of task-specific movement patterns, i.e. dorsiflexion-plantarflexion during the stretching protocol, which matches movement patterns and velocity of the running task (Fletcher and Jones, 2004), might have contributed to the observed improvement in performance. Caution should be exercised when extrapolating the findings of this study to trained male athletes because we only tested male recreational runners. Shellock and Prentice (1985) indicated that elite athletes may need longer warm-ups to prepare appropriately, suggesting that more trained individuals will need a longer warm-up because of their thermoregulatory centre would be more efficient to respond to exercise-generated heat. Additionally, the negative relationship between energy cost and flexibility in men does not seem to be present in female athletes (Beaudoin and Blum, 2005). Furthermore, stiffness values are 29% lower in women compared to men (Granata et al., 2002). As stiffness plays a major role in performance and metabolic cost and is a significant variable affected acutely by stretching, it is conceivable that men and women may respond differently using our measured variables.

According to the spring-mass model system, the combination of passive (tendons) and active (muscles) structures is responsible for the elastic energy recovery during running (Cavagna et al., 1964). Stiffness of the MTU is determined by the relative stiffness of its constituent components, i.e., muscle and tendon. The findings of musculoskeletal modelling studies (Hof et al., 2002; Sasaki and Neptune, 2006) and cadaveric studies (Alexander and Bennet-Clark, 1977; Ker et al., 1987) showed that the lower metabolic energy expenditure by muscle fibres during running was associated with the greater elastic energy stored in the Achilles tendon. Accordingly, we may assume that our protocol may have temporarily increased tendon compliance (Pamboris et al., 2018). During the concentric phase (propulsion phase) of running, the rapid stretching of tendon structures plays a role in lowering the velocity of muscle fibres (Kubo et al., 2000; Kawakami et al., 2002) and having lower stiffness in the plantar flexors after dynamic stretching is suitable for storing higher elastic energy at the stance phase during running, which can further contribute to achieving higher performance. A lower ankle joint stiffness is linked to a lower oxygen cost of running (Struzik et al., 2021).

A number of methodological constraints must be considered when interpreting the results of this study. Oxygen uptake of the whole body was measured during our maximum incremental test and examined dynamic joint and vertical stiffness changes. However, it can be argued that the differences found in the mechanical properties of the muscle-tendon units are caused by dynamic stretching; stiffness may be responsible for the differences on running economy between the two conditions. It was assumed that the triceps surae muscles are the main contributors to the energy expenditure while running. The ankle joint has a crucial role during the stance phase as a joint generator at low running speeds (2.5–3.5 m/s) (Jin and Hahn, 2018; Orendurff et al., 2018), speeds usually performed by recreational runners. It is clear that the ankle joint is important to generate energy in stance phase. Early studies (Winter 1983; Arampatzis et al., 2000) that analysed submaximal running by inverse dynamics reported that the muscles acting around the ankle joint contribute <60% to the total mechanical work during running. Based on these studies, which rely on inverse dynamic analyses, it is reasonable to assume that the ankle joint muscle-tendon unit may be representative of the energy expenditure of submaximal running. It cannot be excluded that individual differences in the moments/forces between participants could exist and could influence the calculated stiffness values.

## Conclusion

Dynamic stretching of the plantarflexors comprising three sets of 20 repetitions at 100 beats/min may enhance running economy in recreational runners, possibly by decreasing joint and vertical stiffness. The effect of dynamic stretching has a short time course, but it can improve the overall running economy. Taken together, these results implied that dynamic stretching could be recommended as part of the warm-up for short duration submaximal running. Future research should focus on optimising the dynamic stretching protocols to influence subsequent stages of performance further and test its implications in other participant groups (i.e., elite athletes, women athletes).

## Data Availability

The raw data supporting the conclusions of this article will be made available by the authors, without undue reservation.

## References

[B1] Albracht K. Arampatzis A. (2013). Exercise-induced changes in triceps surae tendon stiffness and muscle strength affect running economy in humans. Eur. J. Appl. Physiol. 113 (6), 1605–1615. 10.1007/s00421-012-2585-4 23328797

[B2] Alexander R. Bennet-Clark H. (1977). Storage of elastic strain energy in muscle and other tissues. Nature 265 (5590), 114–117. 10.1038/265114a0 834252

[B3] Amrhein V. Greenland S. McShane B. (2019). Scientists rise up against statistical significance. Nature 567, 305–307. Nature Publishing Group. 10.1038/d41586-019-00857-9 30894741

[B4] Arampatzis A. De Monte G. Karamanidis K. Morey-Klapsing G. Stafilidis S. Bruggemann G. P. (2006). Influence of the muscle-tendon unit’s mechanical and morphological properties on running economy. J. Exp. Biol. 209 (17), 3345–3357. 10.1242/jeb.02340 16916971

[B5] Arampatzis A. Knicker A. Bruggemann G. P. (2000). Mechanical power in running: A comparison of different approaches. J. Biomech. 33 (4), 457–463. 10.1016/S0021-9290(99)00187-6 10768394

[B6] Beaudoin C. Blum J. W. (2005). Flexibility and running economy in female collegiate track athletes. J. Sports Med. Phys. Fit. 45 (3), 295–300. 10.1097/00005768-200105001-00128 16230980

[B7] Behm D. Blazevich A. J. Kay A. D. McHugh M. (2015). Acute effects of muscle stretching on physical performance, range of motion, and injury incidence in healthy active individuals: A systematic review. Appl. Physiology, Nutr. Metabolism 41 (1), 1–11. 10.1139/apnm-2015-0235 26642915

[B8] Bishop D. (2003). Warm up II: Performance changes following active warm up and how to structure the warm up. Sports Med. 33 (7), 483–498. 10.2165/00007256-200333070-00002 12762825

[B9] Brughelli M. Cronin J. (2008). A review of research on the mechanical stiffness in running and jumping: Methodology and implications. Scand. J. Med. Sci. Sports 18 (4), 417–426. 10.1111/j.1600-0838.2008.00769.x 18282225

[B10] Butler R. Crowell H. Davis I. (2003). Lower extremity stiffness: Implications for performance and injury. Clin. Biomech. 18 (6), 511–517. 10.1016/S0268-0033(03)00071-8 12828900

[B11] Carter J. Greenwood M. (2015). Does flexibility exercise affect running economy? A brief review. Strength Cond. J. 37 (3), 12–21. 10.1519/SSC.0000000000000127

[B12] Cavagna G. A. Franzetti P. Heglund N. C. Willems P. (1988). The determinants of the step frequency in running, trotting and hopping in man and other vertebrates. J. Physiol. 399 (1), 81–92. 10.1113/jphysiol.1988.sp017069 3404473 PMC1191653

[B13] Cavagna G. A. Saibene F. P. Margaria R. (1964). Mechanical work in running. J. Appl. Physiol. 19 (2), 249–256. 10.1152/jappl.1964.19.2.249 14155290

[B14] Coleman D. Cannavan D. Horne S. Blazevich A. J. (2012). Leg stiffness in human running: Comparison of estimates derived from previously published models to direct kinematic-kinetic measures. J. Biomech. 45 (11), 1987–1991. 10.1016/j.jbiomech.2012.05.010 22682258

[B15] Cramer J. Housh T. J. Johnson G. O. Miller J. M. Coburn J. W. Beck T. W. (2004). Acute effects of static stretching on peak torque in women. J. Strength Cond. Res. 18 (2), 236–241. 10.1519/R-13303.1 15142021

[B16] Di Prampero P. E. (2009). A simple method for assessing the energy cost of running during incremental tests. J. Appl. Physiology 107 (4), 1068–1075. JPEG. 10.1152/JAPPLPHYSIOL.00063.2009/ASSET/IMAGES/LARGE/ZDG0100987650004 19661456

[B17] Farley C. Morgenroth D. (1999). Leg stiffness primarily depends on ankle stiffness during human hopping. J. Biomech. 32 (3), 267–273. 10.1016/S0021-9290(98)00170-5 10093026

[B18] Fletcher I. (2013). An investigation into the effect of a pre-performance strategy on jump performance. J. Strength Cond. Res. 27 (1), 107–115. 10.1519/JSC.0b013e3182517ffb 22395265

[B19] Fletcher I. Anness R. (2007). The acute effects of combined static and dynamic stretch protocols on fifty-meter sprint performance in track-and-field athletes. J. Strength Cond. Res. 21 (3), 784–787. 10.1519/r-19475.1 17685686

[B20] Fletcher I. Jones B. (2004). The effect of different warm-up stretch protocols on 20 meter sprint performance in trained rugby union players. J. Strength Cond. Res. 18 (4), 885–888. 10.1519/14493.1 15574098

[B21] Fletcher I. (2010). The effect of different dynamic stretch velocities on jump performance. Eur. J. Appl. Physiol. 109 (3), 491–498. 10.1007/s00421-010-1386-x 20162300

[B22] Fletcher J. Groves E. M. Pfister T. R. Macintosh B. R. (2013a). Can muscle shortening alone, explain the energy cost of muscle contraction *in vivo*? Eur. J. Appl. Physiol. 113 (9), 2313–2322. 10.1007/s00421-013-2665-0 23712215

[B23] Fletcher J. MacIntosh B. (2017). Running economy from a muscle energetics perspective. Front. Physiol. 8, 433. 10.3389/fphys.2017.00433 28690549 PMC5479897

[B24] Fletcher J. Pfister T. Macintosh B. (2013b). Energy cost of running and Achilles tendon stiffness in man and woman trained runners. Physiol. Rep. 1 (7), e00178. 10.1002/phy2.178 24744857 PMC3970734

[B25] Gelman A. Greenland S. (2019). Are confidence intervals better termed “uncertainty intervals”. BMJ 366, l5381. 10.1136/bmj.l5381 31506269

[B26] Granata K. Wilson S. Padua D. (2002). Gender differences in active musculoskeletal stiffness. Part I.: Quantification in controlled measurements of knee joint dynamics. J. Electromyogr. Kinesiol. 12 (2), 119–126. 10.1016/S1050-6411(02)00002-0 11955984

[B27] Guglielmo L. Greco C. Denadai B. (2009). Effects of strength training on running economy. Int. J. Sports Med. 30 (1), 27–32. 10.1055/s-2008-1038792 18975259

[B28] Hayes P. Walker A. (2007). Pre-exercise stretching does not impact upon running economy. J. Strength Cond. Res. 21 (4), 1227–1232. 10.1519/R-19545.1 18076223

[B29] Herda T. Herda N. D. Costa P. B. Walter-Herda A. A. Valdez A. M. Cramer J. T. (2013). The effects of dynamic stretching on the passive properties of the muscle-tendon unit. J. Sports Sci. 31 (5), 479–487. 10.1080/02640414.2012.736632 23113555

[B30] Hermens H. Freriks B. Merletti R. Stegeman D. Blok J. Rau G. (1999). European recommendations for surface electromyography: Results of the SENIAM project. Roessingh Res. Dev 8, 13–54. 10.1016/S1050-6411(00)00027-4

[B31] Hof A. Elzinga H. Grimmius W. Halbertsma J. P. K. (2002). Speed dependence of averaged EMG profiles in walking. Gait Posture 16 (1), 78–86. 10.1016/S0966-6362(01)00206-5 12127190

[B32] Hopkins W. (2019a). A spreadsheet for bayesian posterior compatibility intervals and magnitude-based decisions. Sportscience 23, 1–3. Available at: http://search.ebscohost.com/login.aspx?direct=true&AuthType=ip.uid.shib&db=s3h&AN=140929944&site=ehost-live&scope=site (Accessed: July 4, 2022).

[B33] Hopkins W. Batterham A. (2018). The vindication of magnitude-based inference. Sportscience 22, 19–29. Available at: http://pacs.sportsci.org/2018/mbivind.htm (Accessed: October 24, 2018).

[B34] Hopkins W. (2019b). Compatibility intervals and magnitude-based decisions for standardized differences and changes in means. Sportscience 23, 1–5. Available at: http://web.a.ebscohost.com/ehost/detail/detail?vid=0&sid=93311832-7d26-4c09-8fd7-dc81ec65b566%40sessionmgr4007&bdata=Jmxhbmc9ZXMmc2l0ZT1laG9zdC1saXZl#AN=140929945&db=s3h (Accessed: July 4, 2022).

[B35] Hopkins W. (2006a). Estimating sample size for magnitude-based inferences. Sportscience 10, 63–70. Available at: http://www.sportsci.org/2006/wghss.htm (Accessed: April 8, 2018).

[B36] Hopkins W. Marshall S. W. Batterham A. M. Hanin J. (2009). Progressive statistics for studies in sports medicine and exercise science. Med. Sci. Sports Exerc. 41 (1), 3–13. 10.1249/MSS.0b013e31818cb278 19092709

[B37] Hopkins W. (2006b). Spreadsheets for analysis of controlled trials, with adjustment for a subject characteristic. Sportscience 10, 46–50. Available at: http://sportsci.org/2006/wghcontrial.htm (Accessed: August 19, 2016).

[B38] Hopkins W. (2015). Spreadsheets for analysis of validity and reliability. Sportscience 19, 36–42. Available at: http://www.sportsci.org/2015/ValidRely.htm.

[B39] Hough P. Ross E. Howatson G. (2009). Effects of dynamic and static stretching on vertical jump performance and electromyographic activity. J. Strength Cond. Res. 23 (2), 507–512. 10.1519/JSC.0b013e31818cc65d 19204571

[B40] Hunter G. Katsoulis K. McCarthy J. P. Ogard W. K. Bamman M. M. Wood D. S. (2011). Tendon length and joint flexibility are related to running economy. Med. Sci. Sports Exerc. 43 (8), 1492–1499. 10.1249/MSS.0b013e318210464a 21266930

[B41] Jin L. Hahn M. E. (2018). Modulation of lower extremity joint stiffness, work and power at different walking and running speeds. Hum. Mov. Sci. 58, 1–9. 10.1016/j.humov.2018.01.004 29331489

[B42] Kawakami Y. Muraoka T. Ito S. KaneHisa H. Fukunaga T. (2002). *In vivo* muscle fibre behaviour during counter-movement exercise in humans reveals a significant role for tendon elasticity. J. Physiol. 540 (2), 635–646. 10.1113/jphysiol.2001.013459 11956349 PMC2290252

[B43] Ker R. Bennett M. B. Bibby S. R. Kester R. C. Alexander R. M. (1987). The spring in the arch of the human foot. Nature 325 (7000), 147–149. 10.1038/325147a0 3808070

[B44] Konrad A. Mocnik R. Nakamura M. Sudi K. Tilp M. (2021). The impact of a single stretching session on running performance and running economy: A scoping review. Front. Physiol. 11, 630282. 10.3389/FPHYS.2020.630282 33551850 PMC7857312

[B45] Kubo K. KaneHisa H. Kawakami Y. Fukunaga T. (2000). Elastic properties of muscle-tendon complex in long-distance runners. Eur. J. Appl. Physiol. 81 (3), 181–187. 10.1007/s004210050028 10638375

[B46] Kubo K. Miyazaki D. Shimoju S. Tsunoda N. (2015b). Relationship between elastic properties of tendon structures and performance in long distance runners. Eur. J. Appl. Physiol. 115 (8), 1725–1733. 10.1007/s00421-015-3156-2 25813019

[B47] Kubo K. Miyazaki D. Yamada K. Yata H. Shimoju S. Tsunoda N. (2015a). Passive and active muscle stiffness in plantar flexors of long distance runners. J. Biomech. 48 (10), 1937–1943. 10.1016/j.jbiomech.2015.04.012 25935690

[B48] Kubo K. Tabata T. Ikebukuro T. Igarashi K. Tsunoda N. (2010a). A longitudinal assessment of running economy and tendon properties in long-distance runners. J. Strength Cond. Res. 24 (7), 1724–1731. 10.1519/JSC.0b013e3181ddf847 20543735

[B49] Kubo K. Tabata T. Ikebukuro T. Igarashi K. Yata H. Tsunoda N. (2010b). Effects of mechanical properties of muscle and tendon on performance in long distance runners. Eur. J. Appl. Physiol. 110 (3), 507–514. 10.1007/s00421-010-1528-1 20535616

[B50] Kyröläinen H. Komi P. (1994). Neuromuscular performance of lower limbs during voluntary and reflex activity in power-and endurance-trained athletes. Eur. J. Appl. Physiol. Occup. Physiol. 69 (3), 233–239. 10.1007/bf01094794 8001535

[B51] Lakens D. Scheel A. M. Isager P. M. (2018). Equivalence testing for psychological research: A tutorial. Adv. Methods Pract. Psychol. Sci. 1 (2), 259–269. 10.1177/2515245918770963

[B52] McMahon T. Cheng G. (1990). The mechanics of running: How does stiffness couple with speed? J. Biomech. 23 (1), 65–78. 10.1016/0021-9290(90)90042-2 2081746

[B53] Moore I. (2016). Is there an economical running technique? A review of modifiable biomechanical factors affecting running economy. Sports Med. 46 (6), 793–807. 10.1007/s40279-016-0474-4 26816209 PMC4887549

[B54] Orendurff M. S. Kobayashi T. Tulchin-Francis K. Tullock A. M. H. Villarosa C. Chan C. (2018). A little bit faster: Lower extremity joint kinematics and kinetics as recreational runners achieve faster speeds. J. Biomech. 71, 167–175. 10.1016/j.jbiomech.2018.02.010 29472010

[B55] Pamboris G. Noorkoiv M. Baltzopoulos V. Gokalp H. Marzilger R. Mohagheghi A. A. (2018). Effects of an acute bout of dynamic stretching on biomechanical properties of the gastrocnemius muscle determined by Shear Wave Elastography. PLoS ONE 13 (5), e0196724. 10.1371/journal.pone.0196724 29723229 PMC5933711

[B56] Pappas P. Paradisis G. P. Exell T. A. Smirniotou A. S. Tsolakis C. K. Arampatzis A. (2017). Acute effects of stretching on leg and vertical stiffness during treadmill running. J. Strength Cond. Res. 31 (12), 3417–3424. 10.1519/JSC.0000000000001777 28118306

[B57] Piaggio G. Elbourne D. R. Pocock S. J. Evans S. J. W. Altman D. G. (2012). Reporting of noninferiority and equivalence randomized trials: Extension of the CONSORT 2010 statement. JAMA - J. Am. Med. Assoc. 308 (24), 2594–2604. 10.1001/jama.2012.87802 23268518

[B58] Powell D. Williams D. S. B. Windsor B. Butler R. J. Zhang S. (2014). Ankle work and dynamic joint stiffness in high- compared to low-arched athletes during a barefoot running task. Hum. Mov. Sci. 34, 147–156. 10.1016/j.humov.2014.01.007 24556475

[B59] Sasaki K. Neptune R. (2006). Muscle mechanical work and elastic energy utilization during walking and running near the preferred gait transition speed. Gait Posture 23 (3), 383–390. 10.1016/j.gaitpost.2005.05.002 16029949

[B60] Schache A. Baker R. (2007). On the expression of joint moments during gait. Gait Posture 25 (3), 440–452. 10.1016/j.gaitpost.2006.05.018 17011192

[B61] Shaw A. Ingham S. A. Fudge B. W. Folland J. P. (2013). The reliability of running economy expressed as oxygen cost and energy cost in trained distance runners. Appl. Physiology, Nutr. Metabolism 38 (12), 1268–1272. 10.1139/apnm-2013-0055 24195628

[B62] Shellock F. Prentice W. (1985). Warming-up and stretching for improved physical performance and prevention of sports-related injuries. Sports Med. 2 (4), 267–278. 10.2165/00007256-198502040-00004 3849057

[B63] Struzik A. Karamanidis K. Lorimer A. Keogh J. W. L. Gajewski J. (2021). Application of leg, vertical, and joint stiffness in running performance: A literature overview. Appl. Bionics Biomech. 2021, 9914278. 10.1155/2021/9914278 34721664 PMC8553457

[B64] Tartaruga M. P. Brisswalter J. Peyre-Tartaruga L. A. Avila A. O. V. Alberton C. L. Coertjens M. (2012). The relationship between running economy and biomechanical variables in distance runners. Res. Q. Exerc. Sport 83 (3), 367–375. 10.1080/02701367.2012.10599870 22978185

[B65] Torres E. Kraemer W. J. Vingren J. L. Volek J. S. Hatfield D. L. Spiering B. A. (2008). Effects of stretching on upper-body muscular performance. J. Strength Cond. Res. 22 (4), 1279–1285. 10.1519/JSC.0b013e31816eb501 18545177

[B66] Winter D. (1983). Moments of force and mechanical power in jogging. J. Biomech. 16 (1), 91–97. 10.1016/0021-9290(83)90050-7 6833314

[B67] Yamaguchi T. Takizawa K. Shibata K. (2015). Acute effect of dynamic stretching on endurance running performance in well-trained male runners. J. Strength Cond. Res. 29 (11), 3045–3052. 10.1519/JSC.0000000000000969 25932984

[B68] Zeni J. Richards J. Higginson J. (2008). Two simple methods for determining gait events during treadmill and overground walking using kinematic data. Gait Posture 27 (4), 710–714. 10.1016/j.gaitpost.2007.07.007 17723303 PMC2384115

[B69] Zourdos M. Bazyler C. D. Jo E. Khamoui A. V. Park B. S. Lee S. R. (2017). Impact of a submaximal warm-up on endurance performance in highly trained and competitive male runners. Res. Q. Exerc. Sport 88 (1), 114–119. 10.1080/02701367.2016.1224294 27636554

[B70] Zourdos M. Wilson J. M. Sommer B. A. Lee S. R. Park Y. M. Henning P. C. (2012). Effects of dynamic stretching on energy cost and running endurance performance in trained male runners. J. Strength Cond. Res. 26 (2), 335–341. 10.1519/JSC.0b013e318225bbae 22266545

